# A Phase II, Randomized, Safety and Immunogenicity Trial of a Re-Derived, Live-Attenuated Dengue Virus Vaccine in Healthy Children and Adults Living in Puerto Rico

**DOI:** 10.4269/ajtmh.14-0625

**Published:** 2015-09-02

**Authors:** Kristen Bauer, Ines O. Esquilin, Alberto Santiago Cornier, Stephen J. Thomas, Ana I. Quintero del Rio, Jorge Bertran-Pasarell, Javier O. Morales Ramirez, Clemente Diaz, Simon Carlo, Kenneth H. Eckels, Elodie Tournay, Jean-Francois Toussaint, Rafael De La Barrera, Stefan Fernandez, Arthur Lyons, Wellington Sun, Bruce L. Innis

**Affiliations:** Viral Diseases Branch, Walter Reed Army Institute of Research, Silver Spring, Maryland; University of Puerto Rico School of Medicine, San Juan, Puerto Rico; Department of Molecular Medicine, La Concepcion Hospital, San German, Puerto Rico; Department of Biochemistry, Ponce School of Medicine, Ponce, Puerto Rico; Centro de Reumatologia Pediatrica de Puerto Rico, San Juan, Puerto Rico; Clinical Research Puerto Rico, Inc., San Juan, Puerto Rico; Department of Molecular Medicine, La Concepcion Hospital, San German, Puerto Rico; Pilot Bioproduction Facility, Walter Reed Army Institute of Research, Silver Spring, Maryland; GSK Vaccines, Wavre, Belgium; GSK Vaccines, King of Prussia, Pennsylvania

## Abstract

This was a double-blind, randomized, controlled, phase II clinical trial, two dose study of re-derived, live-attenuated, tetravalent dengue virus (TDEN) vaccine (two formulations) or placebo in subjects 1–50 years of age. Among the 636 subjects enrolled, 331 (52%) were primed, that is, baseline seropositive to at least one dengue virus (DENV) type. Baseline seropositivity prevalence increased with age (10% [< 2 years], 26% [2–4 years], 60% [5–20 years], and 93% [21–50 years]). Safety profiles of TDEN vaccines were similar to placebo regardless of priming status. No vaccine-related serious adverse events (SAEs) were reported. Among unprimed subjects, immunogenicity (geometric mean antibody titers [GMT] and seropositivity rates) for each DENV increased substantially in both TDEN vaccine groups with at least 74.6% seropositive for four DENV types. The TDEN vaccine candidate showed an acceptable safety and immunogenicity profile in children and adults ranging from 1 to 50 years of age, regardless of priming status. ClinicalTrials.gov: NCT00468858.

## Introduction

Dengue, a mosquito-borne viral infection, has been reported worldwide with increasing frequency since the 1950s.^1^ The World Health Organization (WHO) reported a 30-fold increase in dengue incidence over the past 50 years.^2^ Based on a cartographic modeling approach, it is estimated that approximately 390 million dengue infections occur per year, of which an estimated 96 million results in some degree of illness.^3^

Outbreaks are increasing in endemic regions and are extending into geographical regions that were previously unaffected.^4^–^6^ Although dengue rarely occurs in the continental United States (US), it is endemic in Puerto Rico, the site of this clinical trial. During the period in which this study was conducted, Puerto Rico experienced the largest recorded outbreak of dengue ever reported in the region. During this 2010 epidemic year, there were approximately 21,000 suspected cases of which approximately 15,000 cases were laboratory confirmed. Most infections were due to DENV-1 and DENV-4. Two of the study centers (Caguas and Ponce) were located in the epicenter where most of the cases were reported. The previous major epidemic occurred only 3 years earlier, in 2007, when more than 10,500 cases were reported.^7^

There is no licensed dengue vaccine, although there are many candidates in development. A live-attenuated, tetravalent (four DENV types) dengue virus vaccine candidate (DENV vaccine) was developed by the Walter Reed Army Institute of Research (WRAIR) in collaboration with GSK Vaccines.^8^,^9^ Two phase I/II clinical trials in children and infants in Thailand demonstrated that this WRAIR/GSK candidate DENV vaccine had an acceptable safety profile and elicited immune responses to all four DENV types in over half of the infants and in all of the children after two doses.^10^,^11^ In these early studies, the vaccine was prepared by combining lyophilized monovalent vaccines into a tetravalent preparation at the time of administration.

Subsequently, two phase II, randomized, controlled trials were conducted to evaluate a WRAIR/GSK live-attenuated tetravalent DENV candidate vaccine (TDEN vaccine) prepared from re-derived vaccine strains using three additional passages and lyophilized as a tetravalent product.^12^,^13^ Both trials compared two formulations of the TDEN vaccine against a placebo. One trial was conducted in DENV-naïve adults (i.e., had no previous DENV exposure) and the other in DENV-primed adults. These two trials showed the vaccine to be safe and immunogenic regardless of DENV priming status.

Here we report a larger safety and immunogenicity trial that evaluated the same two TDEN re-derived vaccine formulations versus a saline placebo administered to 636 children and adults ranging from 1 to 50 years of age, in a dengue-endemic region. Safety was evaluated in terms of solicited and unsolicited adverse event (AE) reporting and occurrence of dengue-like illness during the postvaccination period. Immunogenicity was evaluated in terms of neutralizing antibodies elicited to each DENV type.

## Materials and Methods

### Study design.

This was a phase II, randomized, double-blind, placebo-controlled, multicenter, parallel-group clinical trial to evaluate the safety and immunogenicity of two doses of the TDEN vaccine administered 6 months apart (ClinicalTrials.gov NCT00468858).

The clinical trial was conducted at 12 study sites throughout Puerto Rico from 2007 to 2010 in accordance with the provisions of the Declaration of Helsinki, good clinical practice, and U.S. federal regulations. The clinical protocols and supporting documents were approved by the U.S. Army Human Subjects Research Review Board, Office of the Surgeon General. Trial reporting follows the guidelines of the Consolidated Standards of Reporting Trials (CONSORT) and ICH-E3 guidelines.^14^

Prior to the performance of any study-specific procedures, written informed consent was obtained from each adult subject or from the parent/s or guardian/s of young children. Written informed assent was also obtained from children and young adults (7–20 years of age).

### Sponsor and co-development partner.

The study was jointly designed and funded by the Sponsor, the U.S. Army Medical Materiel Development Activity (USAMMDA) and its co-development partner, GlaxoSmithKline Biologicals SA (GSK) and conducted under a U.S. Investigational New Drug (IND) program. The USAMMDA and GSK monitored the conduct of the trial. Investigators collected and encoded the data into a GSK database, and a GSK statistician analyzed the data according to a prespecified and mutually approved plan.

### Vaccines.

The development of the two formulations of the candidate vaccine, including descriptions of the DENV strains, primary dog kidney (PDK), and fetal rhesus lung (FRhL) cell culture passage, and viral concentration have been described previously.^12^ Vaccine potency was confirmed using an immunofocus assay. The TDEN vaccines in this study were formulated to have comparable in vitro potency, except that F19 was planned to contain approximately 10-fold less DENV-4 than F17. In vitro potency tests for the vaccines are shown in Table 1. Note that the actual measured difference in F19 DENV-4 potency was 50-fold less.

The F17 and F19 TDEN vaccines formulations were premixed, freeze dried, and presented in single-dose vials, with sterile water used to reconstitute the vaccine before injection. The placebo, which appeared identical to the vaccines, contained a sterile solution of cell-culture medium, Eagle's minimal essential medium (EMEM), and the same virus stabilizer contained in the vaccine.

After hydration with water for injection, 0.5 mL vaccine or placebo doses were administered subcutaneously into the upper-outer triceps/deltoid area or into the thigh for the youngest children, in accordance with United States Advisory Committee on Immunization Practices (U.S. ACIP) guidelines. Two doses of the TDEN vaccines (F17 and F19) or placebo were to be administered 6 months apart.

### Criteria for study inclusion.

Healthy males and females between 1 and 50 years of age were screened based on medical history, physical examination, and directed cardiac screening and those in general good health were offered enrollment. Children 23 months of age or younger were to have full compliance with the U.S. ACIP-recommended childhood immunization schedule. Subjects were enrolled regardless of pre-vaccination DENV priming status. Female subjects must have been of non-childbearing potential or abstinent or used adequate contraceptive precautions for 30 days prior to vaccination, had a negative pregnancy test within 48 hours prior to vaccination, and agreed to continue such precautions for 60 days after completion of the vaccination series. Exclusion criteria included pregnancy, lactation, history of any neurological disorder or chronic disease, allergies, or reactions likely to be exacerbated by any vaccine component, clinically significant abnormalities, chronic hepatomegaly and/or splenomegaly, cardiac signs or symptoms, and receipt of immune-modifying drug/blood product within 90 days of enrollment. Subjects 12 years of age or older must have had a negative urine pregnancy test and negative human immunodeficiency virus (HIV) screening test, as applicable.

### Randomization and masking.

The study design planned for 720 subjects to be randomly allocated into one of three treatment groups using a block 1:1:1 randomization list generated at GSK from a standard Statistical Analysis System (SAS) program (SAS Institute Inc., Cary, NC). The randomization algorithm used a minimization procedure to balance the number of subjects among treatment by age category (12–23 months, 2–4 years, 5–20 years, and 21–50 years). All subjects enrolled received dose 1 of either F17 or F19 TDEN vaccine or placebo under double-blind conditions (i.e., the investigator and subjects were unaware of the study group assignments) as planned. Dose 2 was also planned to be administered under the same blind, but the protocol was amended so that 19 subjects received dose 2 after they were unblinded (see section “Study design amendment based on potency testing”).

### Clinical safety assessments.

Subjects reported to the study site for six scheduled visits over a 12-month study period for safety and reactogenicity evaluations. Solicited injection site and general AEs were recorded by subjects or their parents/guardians, as applicable, on diary cards for 21 days after each dose. For all subjects, solicited injection site AEs included pain, redness, and swelling, and solicited general AEs included fever, fatigue, headache, abdominal pain, vomiting, diffuse rash on the trunk, and pruritus. Diary cards for subjects 5 years of age and older also included pain behind the eyes, photophobia, nausea, muscle ache, and joint ache. For the subjects 1–4 years of age, irritability/fussiness, drowsiness, and loss of appetite were also recorded.

Investigators also collected AEs spontaneously reported by subjects that occurred over a 31-day follow-up period after each dose. These unsolicited AEs were coded with the use of the *Medical Dictionary for Regulatory Activities*.^15^

Intensities of each AE were scored as grades 1–3, with grade 3 fever defined as an oral body temperature > 39°C/102.2°F and grade 3 redness and swelling defined as > 20 mm diameter around the injection site. All other grade 3 AEs were defined as those preventing normal daily activity. During physical examinations at visits after dose 1 and up to 1 month after dose 2, subjects were assessed for cardiac signs and symptoms using an age-appropriate cardiac algorithm, which included electrocardiograms (ECGs). Abnormal results triggered a consultation with a cardiologist. Evaluation plans were designed and determined by the evaluating physician based on the presenting symptom complex. There were no constraints on the diagnostic methods that could be used. This evaluation was performed to address the theoretical potential for a live-attenuated DENV vaccine to cause AEs similar to the atypical and infrequent cardiac manifestations reported after wild-type DENV infections.^16^

Investigators also recorded any serious adverse events (SAEs), defined as medically significant events including those resulting in hospitalization, disability, or death, throughout the study.

Measures taken to minimize AE reporting bias included the use of standardized diary cards and physical examinations, study personnel training, and careful monitoring of all subjects by study nurses at each study site, in addition to conducting the study in an observer-blind, randomized manner.

Throughout the study, subjects were evaluated by an investigator if they developed high fever (oral temperature > 39°C/102.2°F) or if any fever ≥ 38°C/100.4°F (oral temperature) was measured at least once on two successive days. Subjects/caregivers were then instructed to monitor the fever and associated symptoms for an additional day. If an additional day of fever occurred, the subject was asked to return to the investigator for evaluation and a blood draw for DENV viremia, if there was no other evident diagnosis. A confirmed dengue case met the following criteria: 1) oral temperature ≥ 38°C/100.4°F measured at least once on 3 successive days and no alternative diagnosis could be made with reasonable certainty by the physician and 2) DENV viremia was found. DENV RNA, as a measure of viremia, was determined using a reverse transcriptase quantitative polymerase chain reaction (RT-qPCR).^17^

The limits of detection (LOD) for the RT-qPCR assay expressed in log genome equivalents/mL (log Geq/mL) were 3.8 log Geq/mL (DENV-1), 5.2 log Geq/mL (DENV-2), 3.1 log Geq/mL (DENV-3), and 4.5 log Geq/mL (DENV-4). A test for dengue viremia was only performed on acute blood specimens from subjects with suspected dengue.

### Study design amendment based on potency testing.

During the study, a routine vaccine stability test revealed a decrease below the preestablished potency specifications for the F19 vaccine. At the time of this discovery, some subjects had not received their second dose of vaccine. Nineteen subjects who were due to receive the second F19 vaccine dose were unblinded and consequently excluded from the according to protocol (ATP) cohort for immunogenicity. After re-consenting, they were administered in an open manner their second dose of F19 vaccine from a new batch (but same lot) of frozen vaccine with acceptable potency. Subjects in the other two groups remained under double blind through the end of the study.

### Evaluation of immune response.

To characterize TDEN vaccine immunogenicity and preexisting dengue antibody status, anti-DENV neutralizing antibodies to each DENV type were measured on blood samples collected at each of the following study visits: on the days of vaccination (Day 0 and Month 6 visits), Month 3 visit for the 3 months post-dose 1 evaluation, and Month 7 visit for the 1 month post-dose 2 time point. The Translational Medicine Branch, Pilot Bioproduction Facility of WRAIR conducted the analyses using a quantitative microneutralization assay (MN50) performed in Vero cells, as previously described.^12^ Seropositivity was defined as a titer ≥ 1:10 dilution.

This MN50 assay was used previously in a study among U.S. subjects receiving these same vaccine formulations in which it was shown to be specific and sensitive for the detection of anti-DENV neutralizing antibodies (e.g., limit of the blank < 1:3.3; limit of detection < 1:7, and limit of quantification ≤ 1:10 for all four serotypes).^12^ The precision of the assay was estimated to range from 39% to 59% CV (coefficient of variance) depending on serotype.^12^

### Statistical analyses.

#### Safety.

The primary safety analyses were performed on the total vaccinated cohort (TVC), that is, all subjects who received at least one dose and for whom data were available. Analyses were stratified by pre-vaccination DENV priming status (unprimed, primed) and age category (younger than 5 years, 5–17 years, and 18–50 years).

An unprimed subject was defined as having DENV neutralizing antibody titer < 1:10 for all four DENV types and a primed subject was defined as having DENV neutralizing antibody titer ≥ 1:10 for at least one DENV type.

The overall percentages of subjects reporting an AE after vaccine administration (21 days after each vaccination for solicited AEs and 31 days after each vaccination for spontaneously reported unsolicited AEs) were tabulated with exact 95% confidence intervals (CIs), by type and intensity (any grade and grade 3) for each age category, priming status, and group. All SAEs occurring during the study were listed. The proportion of subjects with abnormal dengue physical examination findings detected up to 31 days after each vaccination was tabulated with exact 95% CI.

#### Immunogenicity cohort for analysis.

The primary analyses of immunogenicity were performed on the ATP cohort for immunogenicity (i.e., subjects who met all eligibility criteria, followed protocol procedures, and for whom data were available for at least one immunogenicity endpoint). This cohort was adjusted based on results of stability testing of the vaccine lots that was conducted as a result of the vaccine potency issue previously described.

The stability testing affected which subjects would be included into the ATP cohort for the F19 group. In vitro testing of F19 vaccine administered at dose 1 indicated no loss of potency up to 6 months, but tests at 9, 12, and 15 months of refrigerator storage indicated loss of potency in one of nine samples tested (11%). However, in vitro testing at months 18, 21, and 24 indicated loss of potency in six of nine samples tested (67%). Therefore, F19 dose 1 administration fell under one of three categories depending on which month the subjects received their vaccination: after the vaccine had been stored ≤ 6 months; > 6–15 months; or > 15 months. Loss of potency was then evaluated by measuring the effect of “time of vaccine storage” on antibody titers at study Month 7 (i.e., 1 month post-dose 2) for unprimed subjects, to determine whether titers were lower for subjects vaccinated with a vaccine stored more than 6 months or more than 15 months.

The most sensitive indicator of vaccine loss of potency was considered to be the DENV-4 geometric mean antibody titer (GMT) in unprimed subjects. The data showed no significant difference in DENV-4 GMT in unprimed subjects for those vaccinated with F19 vaccine stored 6 months or less and those vaccinated with F19 vaccine stored between 6 and 15 months. Therefore, subjects who received a F19 dose stored between 6 and 15 months were not eliminated from the ATP cohort for immunogenicity analysis. Subjects who received F19 stored longer than 15 months, however, were eliminated from the ATP cohort.

#### Immunogenicity analyses.

The following immunogenicity parameters were calculated by group at each testing time point: seropositivity rate and tetravalent response rate, both calculated with exact 95% CIs and GMT for each DENV type calculated with 95% CIs. Seropositivity rate per DENV type was defined as the percentage of subjects with DENV neutralizing antibody titer ≥ 1:10 dilution (referred to as 1:10) and tetravalent response rate was defined as the proportion of subjects with antibody titers ≥ 1:10 for all four DENV types, at any given time point. GMTs were calculated by taking the antilog of the mean of the log titer transformations, with a value of 5 given to titers < 1:10 and a value given at 2,430 for titers > 1:2,430 (maximum limit of detection). Immunogenicity analyses were stratified by priming status. Pre-vaccination priming status was determined for the following age categories: 12–23 months, 2–4 years, 5–20 years, and 21–50 years.

Analysis of variance (ANOVA) was performed on log_10_ transformation of the titers for each type to compare dengue vaccine formulations F17 and F19 over all age groups in unprimed subjects. The two formulations were considered significantly different for one type if the *P* value was less than or equal to 0.0125 (the Bonferroni correction was applied to address the problem of multiple comparisons). The sample size was selected to offer 83% power to detect a 2-fold difference in GMT among subjects seronegative at baseline, assuming a standard deviation of 0.7 for the log titers and that there would be at least 130 evaluable baseline naive subjects per treatment group (F17 and F19).

An exploratory regression analysis was performed to assess the effect of age on vaccine response to DENV at study Month 7 (i.e., 1 month after dose 2) adjusted by sex, treatment group, and pre-vaccination seropositivity (negative/positive), for each DENV type. Vaccine response for unprimed subjects was defined as a postvaccination increase in neutralizing antibody titer above the assay cutoff. For initially primed subjects, a vaccine response was defined as having at least a 4-fold increase in neutralizing antibody titer postvaccination compared with baseline titer. With a maximum limit of detection of 2,430 (1:10); subjects who had a titer greater than 607.5 at baseline could not be confirmed as having a vaccine response (approximately 50 subjects per group and per DENV type) and were excluded from analysis.

## Results

### Study population.

#### Enrollment.

**Figure 1.**
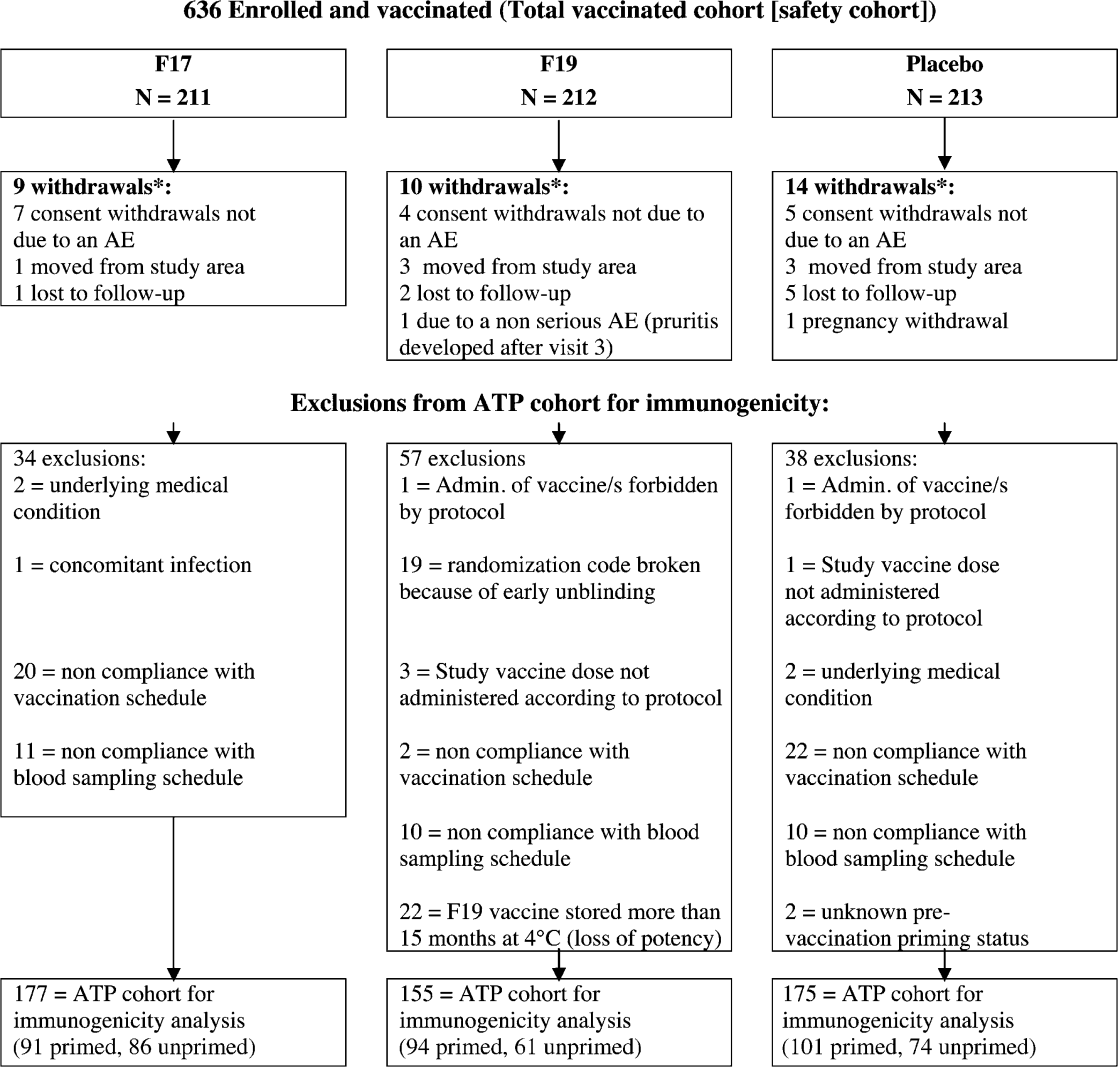
Subject disposition. * Withdrawals through study Month 12. *N* = number of subjects; AE = adverse event; ATP = according to protocol.

Of the 636 subjects enrolled (211, 212, and 213 in F17, F19, and placebo groups, respectively), 603 completed the study. Figure 1 shows the subject disposition. Thirty-three subjects were withdrawn before the Month 12 visit, mainly because they were lost to follow up, moved from the area, or withdrew consent. One withdrawal was due to a nonserious AE (pruritus, last visit Month 3, F19 group) and one because of a pregnancy (subject in placebo group followed until delivery and was in good condition; no problems with the birth secondary to vaccination). None of the subjects were withdrawn because of a SAE.

In the F19 group, 22 out of 212 subjects had already received a second dose of F19 DENV that had been stored at refrigerator temperature for at least 15 months and had possible reduced potency, and therefore, were excluded from immunogenicity analyses. In addition, the 19 subjects who were unblinded for their second dose of F19 vaccine were also excluded from immunogenicity analyses.

The study was conducted between July 2007 and April 2010 in sites throughout Puerto Rico. At the time of recruitment, there was a shortage of *Haemophilus influenzae* type B vaccine in Puerto Rico, which affected enrollment of the lowest age subset, that is, children under the age of 2 years because full compliance with routine childhood vaccinations was an inclusion criterion for this age group. Therefore, the number of the youngest subjects enrolled (*N* = 96) reached slightly more than half the planned target of 180.

At least 95% of the subjects in each group received both doses of study vaccine/placebo (609 subjects received 2 doses; 27 subjects received only 1 dose [8, 10, and 9 subjects in the F17, F19, and placebo groups, respectively]). A diary card was completed by at least 99% of subjects in each group and returned after each vaccine/placebo dose administered.

#### Baseline characteristics.

The demographic profiles of the three treatment groups were comparable with respect to mean age, gender, ethnicity, and race. Among the three groups, the overall mean age was 13.4 years and 52.7% of subjects were female. Most enrolled subjects (77.6%) were Puerto Rican.

### Safety results.

#### Overall reporting of solicited AEs.

During the two 21-day follow-up periods postvaccination, 65.7–72.0% of subjects across the three groups reported at least one solicited injection site reaction or general AE as shown in Table 2. There were no major differences in terms of occurrence of any injection site AE or any solicited general AEs observed in primed and unprimed subjects. The percentage of unprimed subjects who reported at least one solicited AE after either dose ranged from 61.1% to 75.0% per group whereas the percentage for primed subjects ranged from 66.1% to 69.5% per group. Most solicited symptoms were transient and overall symptom reporting did not increase from dose 1 to dose 2 for any group (data not shown).

Most solicited AEs reported were graded as mild to moderate: at least one grade 3 solicited AE was reported by 9.0–10.4% of subjects across the three groups with no major differences among primed and unprimed subjects (data not shown).

#### Individual solicited AEs reported.

The incidence of each injection site reaction (pain, redness, and swelling), overall per subject is presented in Table 3, and the incidence reported per group by priming status is shown in Supplemental Figure 1.

Injection site pain was the most frequently reported reaction during the 21 days following both vaccinations in all groups (19.5–25.1% of subjects across the three groups). Most solicited injection site AEs lasted 2 days or less and were mild to moderate in severity. The most frequently reported grade 3 reaction was redness (maximum of 2.4% of subjects for any group). There were no notable differences regardless of priming status between F17 and F19 groups in terms of the frequency of any injection site symptom (Supplemental Figure 1).

The incidences of solicited general AEs (overall per subject) reported during the 21-day postvaccination periods is provided in Table 4 for the seven general AEs common to all age groups (abdominal pain, fatigue, fever, headache, pruritus, rash, and vomiting).

Fever was the most frequently occurring solicited general AE reported overall (33.3–39.1% of subjects across the three groups) followed by headache reported by 27.6–30% of subjects across the three groups.

The most frequently reported grade 3 general AE was fever (3.8–6.2% of subjects) followed by grade 3 headache (0.5–2.9% of subjects), across the three groups. All other solicited grade 3 general AEs were reported with a very low incidence (≤ 0.5% of subjects, per group).

The incidence of general AEs per treatment group based on priming status is depicted in Supplemental Figure 2. Fever was the most frequently occurring solicited general AE reported among the initially unprimed subjects; whereas fever and headache were most frequently reported among the initially primed subjects. There were no clinically noteworthy differences between the initially unprimed and primed subjects in each group with regard to the incidence of each general AE with the possible exception of headache, which appeared to be more frequent among the initially primed subjects, most notably in the placebo group.

The incidence of solicited general AEs by age categories is depicted in Supplemental Figure 3. Among the 1- to 4-year-old subjects, drowsiness, irritability, and loss of appetite were also solicited, in addition to the AEs recorded/solicited for all subjects. Among subjects 5 years of age and older, the additional solicited AEs included arthralgia, muscle aches, nausea, pain behind the eyes, and photophobia. Fever followed by loss of appetite was the most frequently reported solicited general AE among the younger age groups with an incidence below 40% in any group. Fever followed by headache was the most frequently reported solicited general AEs among subjects 5–17 years of age with fever reported by more than 60% of subjects in the F17 group. Among the adult population (subjects 18 years of age and older), headache was the most frequently reported solicited general AE with the highest incidence observed in the placebo group (50% of subjects).

#### Spontaneous reporting of unsolicited AEs.

Among children 1–4 years of age, at least one unsolicited AE was reported by 57.8% (F17), 64.5% (F19), and 69.9% (placebo) of subjects in the 31-day period after both vaccinations (data not shown). The most frequently reported unsolicited AEs in this age category were upper respiratory tract infection (13.3% [F17], 22.6% [F19], and 22.6% [placebo]) and nasopharyngitis (14.4% [F17], 6.5% [F19], and 17.2% [placebo]). In this younger age group, two subjects (2.2%) reported a grade 3 unsolicited AE in the F17 group (conjunctivitis, diarrhea), four (4.3%) in the F19 group (diarrhea, thirst, pneumonia, and crying), and three (3.2%) in the placebo group (gastroenteritis, pharyngitis, and asthma). All grade 3 unsolicited AEs were considered by the investigator as unrelated to vaccination except for the one incidence of crying in a 3-year-old subject.

Among subjects 5–50 years of age, at least one unsolicited AE was reported by 36.4%, 33.6%, and 33.3% of subjects in the F17, F19, and placebo groups, respectively, with the most frequently reported unsolicited AE being upper respiratory tract infection (5.8–6.7% of subjects across the three groups). All other AEs were reported by fewer than 5% of subjects per treatment group. Grade 3 unsolicited AEs were rare (one subject each [0.8%] pharyngitis in the F19 and humerus fracture in the placebo groups, and none in the F17 group), and unrelated to vaccination.

#### Reported SAEs and suspected dengue cases.

SAEs were reported by eight (8.9%), four (4.3%), and eight (8.6%) subjects in the F17, F19, and placebo groups, respectively, among those in the 1–4 years of age category. SAEs among this younger age group included cellulitis, conjunctivitis, sinusitis, upper respiratory tract infection, pneumonia, tonsillitis, viral infection, bronchitis, gastroenteritis, and diarrhea. Among subjects 5–50 years of age, three subjects (all in the placebo group) reported SAEs including gastroenteritis, humerus fracture, and renal/urinary disorders. None were considered to be related to vaccination. There were no fatalities reported during this study.

Throughout the study period, 19 cases of suspected dengue (oral temperature ≥ 38°C/100.4°F measured at least once on 3 successive days and no alternative diagnosis) were reported for 18 subjects none of which were confirmed as dengue. Eight cases occurred during the 31 days postvaccination period (six after dose 1 and two after dose 2), including 3–4 cases in each TDEN group and 1 in the placebo group. Eleven of the 19 cases occurred more than 31 days after a vaccine/placebo dose (four cases each in the TDEN groups and three in the placebo group). All 19 cases received medical attention without hospitalization. There were no subjects with 3 consecutive days of fever and a positive test for dengue viremia (DENV RNA).

#### Cardiac evaluations.

There were no findings consistent with a cardiac lesion reported in the study. Fifteen children 1–4 years of age (five in the F17 group, six in the F19 group, and four in the placebo group) had a cardiac finding on physical examination that led to a directed cardiopulmonary examination. All findings were normal: no subject had peripheral edema, periorbital edema, tachypnea, rales, or hepatomegaly. Six subjects 5–50 years of age (three in the F17 and two in F19 groups, and 1 in placebo group) had a cardiac finding on physical examination that led to an ECG. No findings from the ECGs were consistent with a cardiac lesion and all findings from the cardiopulmonary examinations were normal: no cases of peripheral edema, periorbital edema, tachypnea, rales, or hepatomegaly were identified. One 8-year-old subject in the placebo group was assessed by a cardiologist after an abnormal ECG finding, whereupon the cardiologist confirmed that the abnormal finding was not consistent with a cardiac lesion.

### Immunogenicity results.

#### Pre-vaccination priming status.

**Figure 2.**
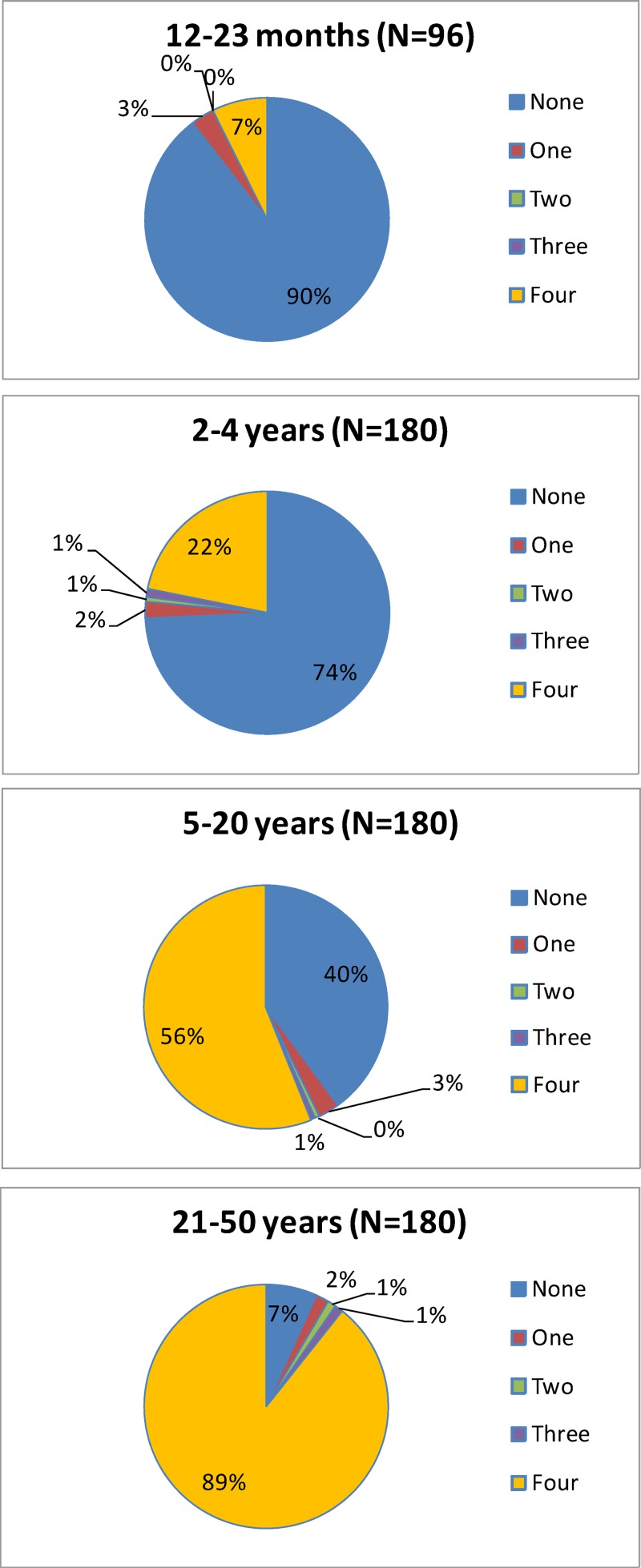
Pre-vaccination priming status, by age (total vaccinated cohort [TVC]).

The percentage of subjects in the ATP cohort categorized as being primed to DENV (i.e., seropositive to at least one DENV type) at baseline was 51.4%, 60.6%, and 57.7% for the F17, F19, and placebo groups, respectively. Figure 2 shows the distribution of pre-vaccination DENV priming status by age category. Most of the subjects younger than 5 years of age were unprimed to any DENV type prior to receiving the first dose of vaccine or placebo (89.6% of the 12 to 23-month-old subjects and 73.9% of the 2 to 4-year-old subjects). Conversely, the majority of subjects 5 years of age and older were primed to all four DENV types (56.1% of the 5- to 20-year-olds and 88.9% of the 21- to 50-year-olds).

Immunogenicity results are presented based on baseline priming status. Seropositivity rates for each DENV type are presented in Table 5. Note that post-dose 1 Month 6 data were similar to post-dose 1 Month 3, at all time points and for each group. Therefore, only data for Month 3 are shown.

#### Unprimed subjects.

Three months after dose 1, seropositivity rates among unprimed subjects were 20.5% and 26.7% for DENV-1, 26.5% and 43.3% for DENV-2, 14.5% and 21.7% for DENV-3, and 37.3% and 26.7% for DENV-4 for F17 and F19 groups, respectively. One month post-dose 2, seropositivity rates for all DENV types increased substantially in the TDEN groups, ranging between 89% and 97.6% of subjects in the F17 group and between 78.0% and 93.2% in the F19 group (Table 5).

Among unprimed subjects in the TDEN groups, the tetravalent response rate was ≤ 20% 3 and 6 months post-dose 1, and increased to 86.6% (F17 group) and 74.6% (F19 group) 1 month post-dose 2 (data not shown).

**Figure 3.**
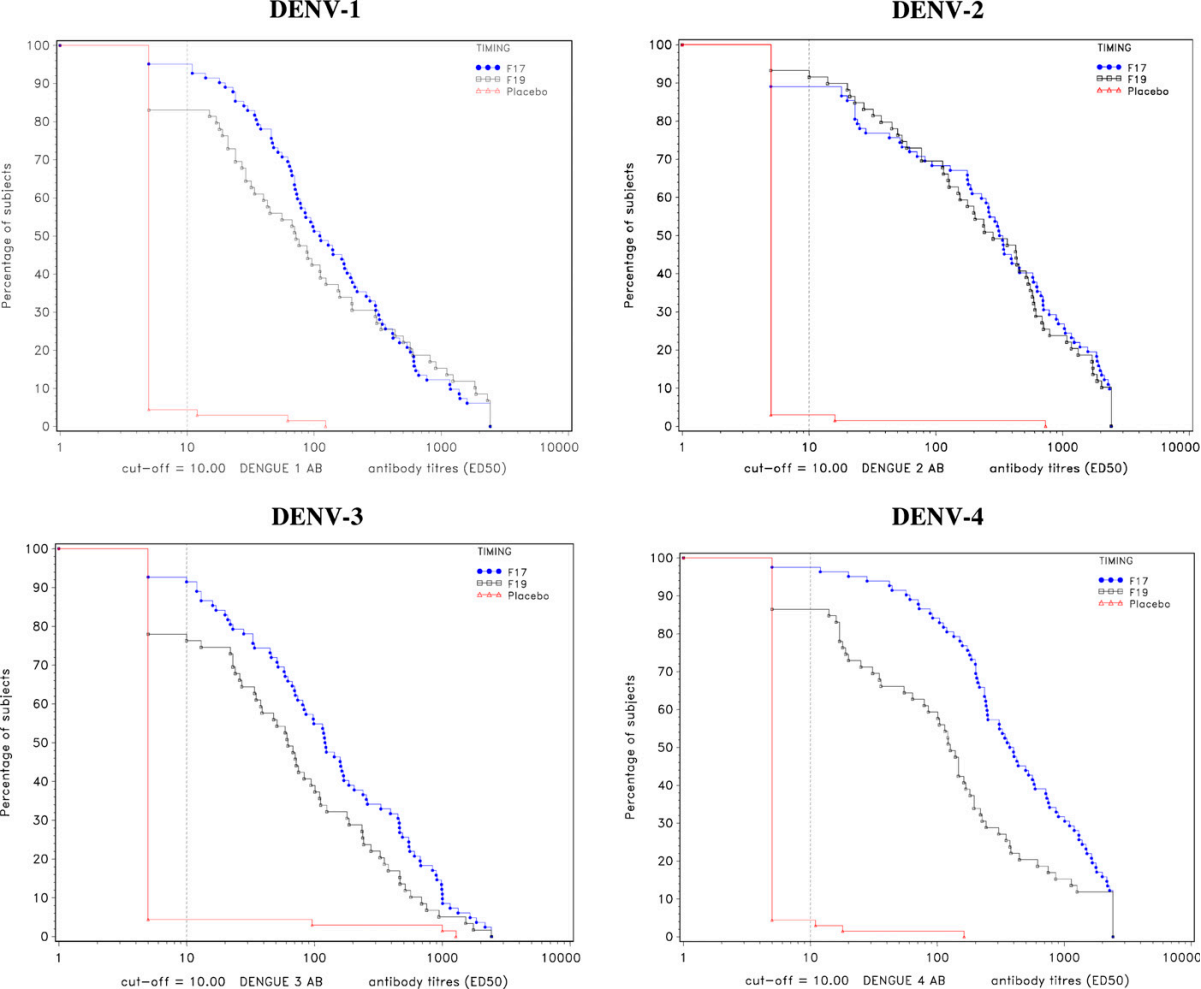
Reverse cumulative distribution curve for dengue virus (DENV) antibody titers at Month 7 for unprimed subjects (according to protocol [ATP] cohort for immunogenicity).

GMTs for unprimed subjects are presented in Table 6 and the distribution of antibody titers is presented in reverse cumulative curves in Figure 3

. In general, GMTs 3 months after dose 1 were low, ranging between 7.2 and 26.3, for each DENV type in both TDEN groups. One month after dose 2, an increase of GMT was observed in TDEN groups with GMTs ranging from 124.5 to 377.7 in the F17 group and from 60.3 to 215.2 in the F19 group. GMTs were significantly higher in F17 compared with F19 for DENV-4 (377.7 versus 105.9, *P* < 0.0001). Although not statistically significant, GMTs also tended to be higher in F17 compared with F19 for DENV-1 (130.9 versus 84.4, *P* = 0.143) and DENV-3 (124.5 versus 60.3, *P* = 0.016), whereas similar GMTs were observed between groups for DENV-2 (218.6 versus 215.2, *P* = 0.962. GMTs in the placebo group were low for each DENV type (subjects attained a maximum GMT of 6.1 for any given DENV type). The reverse cumulative curves show that while titer distributions were similar for anti-DENV-2, the distributions appeared to be marginally in favor of F17 over F19 for postvaccination antibody responses to DENV-1, DENV-3, and DENV-4.

#### Primed subjects.

**Figure 4.**
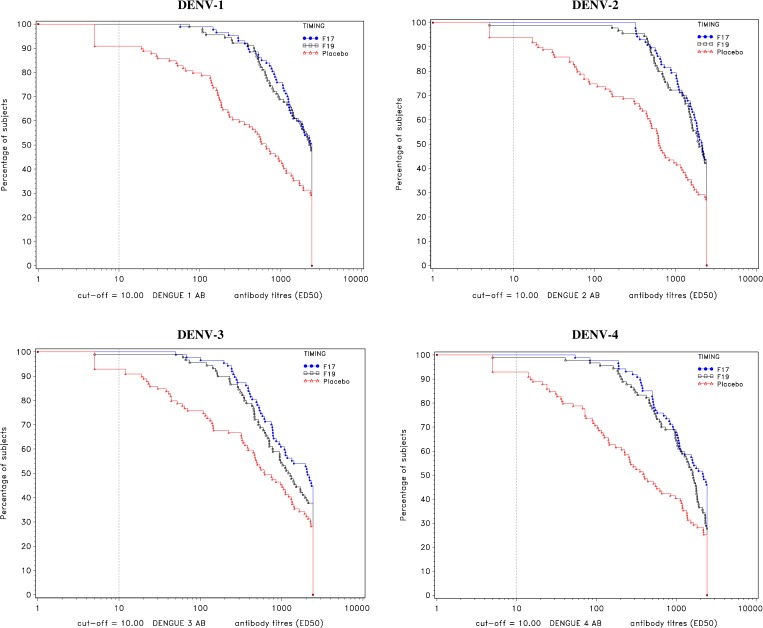
Reverse cumulative distribution curve for dengue virus (DENV) antibody titers at Month 7 for primed subjects (according to protocol [ATP] cohort for immunogenicity).

Among the primed subjects, seropositivity rates were similar at each time point. For each of the two TDEN groups and each DENV type, seropositivity rates ranged from 92.6% to 98.9% at pre-vaccination and ranged from 98.9% to 100% 1 month after dose 2 (Table 5). GMTs for DENV antibodies at pre-vaccination, 3 months post-dose 1, and 1 month post-dose 2 for primed subjects are presented in Table 7, and reverse cumulative distribution for DENV antibody titers 1 month post-dose 2 is shown in Figure 4. For both TDEN vaccine groups and for each DENV type, GMTs increased primarily from pre-vaccination (324–564) to post-dose 1 (1,042–1,590). Post-dose 2 GMTs were 901–1,514. The reverse cumulative distribution curves illustrate the similarity of F17 and F19 postvaccination antibody responses for all DENV types.

#### Effect of age on immune response.

A logistic regression analysis revealed that there was no effect of age category (12–23 months/2–4 years/5–20 years/21–50 years) on vaccine responses to any of the four DENV types 1 month post-dose 2 after adjusting for sex, treatment group, and seropositivity at baseline (*P* = 0.78, *P* = 0.40, *P* = 0.52, and *P* = 0.45 for DENV-1, DENV-2, DENV-3, and DENV-4, respectively).

## Discussion

This study was the third in a series of phase I/II clinical assessments of the re-derived WRAIR–GSK TDEN live-attenuated vaccine—the first two involving healthy adults, both primed and unprimed to dengue virus. The present study was the first to also evaluate the re-derived dengue vaccine candidate in children and enabled us to evaluate the effect of DENV priming on vaccine immunogenicity and safety in both children and adults concurrently.

The vaccine formulations F17 and F19 used in this study were also used in two previous studies.^12^,^13^ They were tested for stability after 22 months of storage and prior to beginning the clinical study. The F17 formulation titers for DENV-1, DENV-2, DENV-3, and DENV-4 were stable and did not vary beyond the variability of the immunofocus assay (3-fold). However, for the F19 formulation, there was a loss of infectivity from 3- to 10-fold with DENV-4 having the most significant titer loss (10×) when compared with the potency at release and also when previously used.^12^,^13^ The loss of potency in representative samples of vaccine held centrally for stability monitoring was not related to a cold chain issue, as the storage conditions were controlled and monitored. Stable potency for live vaccines depends on the formulation (including a stabilizer) and the quality of lyophilization. Some U.S.-licensed live viral vaccines have 18 months or more shelf life before expiry; others have less. For the vaccine candidate under evaluation, the experience in this trial suggests that the formulation and lyophilization process could be targeted for improvement. Given the lack of an established correlate of protection, the level of immune response that equates to protection is unknown. Consequently, a minimum level of potency required for protection has not been established. Therefore, we cannot infer whether this decrement in potency is associated with any change in theoretical efficacy, nor can we infer any safety implications.

Our results support those of the previous two studies that showed the TDEN vaccine formulations were generally well tolerated by healthy adults whether they had prior priming with dengue virus.^12^,^13^ Common to all three studies, most injection site symptoms were transient and mild to moderate in severity, there were few reports of grade 3 AEs overall, and injection site reactions did not increase with administration of the second dose. In the adult trial conducted in Thailand with mostly primed adults, the F19 formulation seemed to have a more favorable profile than the F17 formulation in terms of injection site pain and general symptoms, although, it should be noted that the sample size was too small for a comparative analyses to be made.^13^ In the adult trial conducted in the United States with mostly unprimed subjects, no differences in the safety profiles of the F17 and F19 TDEN vaccines were observed.^12^ In the current study, we also did not observe any notable differences in the two vaccine formulations with regard to the safety and reactogenicity profiles. Although the study was not powered to detect a statistical difference between groups with regard to AE reporting, we did not observe any notable differences in the occurrence of injection site reactions, solicited general AEs, and unsolicited AEs, reported among the TDEN and placebo groups, and the AE profiles were similar regardless of priming status. There were no vaccine-related SAEs reported and no fatalities.

Cardiac evaluation was performed in all three studies of the TDEN vaccine to explore whether replication of the vaccine viruses was potentially associated with cardiac AEs, a manifestation reported after a wild-type DENV infection, as reported in a case study and literature review by Lee and others.^16^ In the case study, dengue hemorrhagic fever, complicated by acute myocarditis and pulmonary edema, was reported in a 65-year-old woman infected with DENV-3. The patient recovered 3 days later. The author's literature search found 18 pertinent articles involving 339 dengue-affected patients with cardiac complications that varied considerably, from self-limiting tachy-brady arrhythmia to severe myocardial damage, leading to hypotension and pulmonary edema and rarely a fatal outcome was reported in some cases of dengue with cardiac complications. There were no reports of cardiac AEs in our study, in line with the previous two adult TDEN vaccine studies. Based on these negative findings in almost 500 TDEN-exposed subjects, we believe that specific monitoring for cardiac lesions in any future evaluation of this vaccine is unwarranted.

Most of the younger subjects in this study had no antibody titers detected for any of the DENV types prior to their first vaccine dose. Among subjects younger than 2 years of age, and subjects 2–4 years of age, 89.6% and 73.9%, respectively, had no antibody titers to any DENV type. However, 60.0% of subjects 5–20 years of age and 92.8% of subjects 21–50 years of age had antibody titers to at least one of the DENV types prior to vaccination, with for most of them a tetravalent profile. This observation was expected and is consistent with the endemic transmission of all four DENV types in Puerto Rico for several decades.

GMTs for each DENV type among unprimed subjects in both vaccine groups increased from 3 months post-dose 1 to 1 month post-dose 2. One month post-dose 2 GMTs in the F17 group reached 124.5–377.7, whereas GMTs for the F19 group reached 60.3–215.2 depending on DENV type. The GMTs were statistically significant higher in the F17 compared with F19 group only for DENV-4 (*P* < 0.0001), which suggests that a reduction in the vaccine's DENV-4 content could reduce the homologous immune response. The higher GMTs for F17 formulation linked to an approximate 50-fold higher DENV-4 content per dose suggests that achieving a consistent immunogenicity profile with this vaccine may require precise control of each viral component's potency in the tetravalent blend, both at release and at the end of a shelf life as validated by a clinical trial using new and aged vaccine lots. One month after the second dose of the TDEN vaccine formulations, GMTs appeared to be 3–10 times higher in primed subjects (mostly adults) compared with unprimed subjects (mostly children) depending on DENV type and group. Although no obvious differences were observed between F17 and F19 among primed subjects, GMTs among primed subjects in the TDEN groups were noticeably higher than in the placebo group. In this study, we found no effect of age on immune responses to any of the four DENV types after adjusting for sex, treatment group, and seropositivity at baseline.

In the current study, DENV exposure prior to vaccination was measured by testing DENV antibody responses to each type. Most primed subjects in Puerto Rico were likely to have had DENV exposure, although the contribution of other flaviviruses or vaccination cannot be excluded (St. Louis Encephalitis virus, West Nile virus, yellow fever vaccination, or Japanese encephalitis vaccination).

As previously reported for these vaccine formulations, a single dose of TDEN vaccine was shown to be immunogenic in primed subjects, regardless of formulation, although antibody titers appeared to be declining, from Month 3 to Month 7 of the study for the F19 formulation. Two doses elicited an immune response to all four DENV types in unprimed subjects.

DENV antibodies at or above the assay positive threshold in previously negative subjects were assessed to indicate activation of an adaptive immune response associated with recovery from infection with one or more of the vaccine viruses. Previously seropositive subjects were assessed by measuring an increase in each DENV antibody type and whether primed subjects with less than tetravalent antibody responses were promoted to acquire tetravalent neutralizing antibodies. As has been the case in the two previous studies,^12^,^13^ most unprimed vaccine recipients failed to develop tetravalent responses after the first vaccine dose yet both TDEN vaccine formulations elicited moderate immunogenicity responses across all DENV types after two doses. It should be noted that the level of neutralizing antibodies measured by the WRAIR MN50 test, which is correlated with immunity to dengue, is unknown. Low levels of neutralizing antibody to DENV may not be predictive of immunity. Therefore, the efficacy of this vaccine candidate can only be determined in a clinical endpoint trial.

During routine testing of stored vaccine samples, we noted a loss of vaccine potency for the F19 TDEN formulation. Despite the measured potency loss in the stored TDEN F19 vaccine, there was no significant difference in DENV-4 GMT in unprimed subjects for those vaccinated with F19 vaccine formulation stored either ≤ 6 months or 6–15 months and no decrease in the immune response was observed in subjects who received a second dose of TDEN F19 vaccine that was potentially compromised. However, the number of subjects affected was too small to reliably detect modest reductions in immunogenicity.

## Conclusion

In this phase II study, the WRAIR–GSK re-derived, live-attenuated, tetravalent TDEN vaccine candidate was found to have a clinically acceptable safety and immunogenicity profile in DENV-unprimed and DENV-primed children and adults ranging from 1 to 50 years of age. Two doses of these vaccine candidates were immunogenic in DENV-naïve subjects, with F17 having a better overall immunogenicity profile compared with F19. A single dose of TDEN was shown to be immunogenic in primed subjects, regardless of formulation.

The decision to extend the development of this live-attenuated viral dengue vaccine will depend in part on the evidence that it elicits consistent, potent, and durable humoral immunity. In this study, we noted that achieving consistent immunogenicity between different lots may be challenging because modest differences in DENV-4 titer between F17 and F19 candidates at the time of formulation and release resulted in potentially important differences in immune response when subsequently administered to subjects. A long-term follow-up study of children previously vaccinated in Thailand with an earlier version of this vaccine has been conducted and will be the subject of a future report to provide information regarding antibody persistence and safety.

## Supplementary Material

Supplemental Figures.

## Figures and Tables

**Table 1:** TDEN vaccine formulations and placebo

TDEN vaccine formulation F17	TDEN vaccine formulation F19
DENV-1: 4.5 log_10_ FFU/mL	DENV-1: 4.0 log_10_ FFU/mL
DENV-2: 4.9 log_10_ FFU/mL	DENV-2: 4.6 log_10_ FFU/mL
DENV-3: 4.6 log_10_ FFU/mL	DENV-3: 4.1 log_10_ FFU/mL
DENV-4: 5.1 log_10_ FFU/mL	DENV-4: 3.4 log_10_ FFU/mL
Viral origin and manufacturing:	
DENV-1: 45AZ5, PDK 27	
DENV-2: S16803, PDK 50	
DENV-3: CH53489, PDK 20	
DENV-4: 341750, PDK 6	
Placebo	
A sterile solution of EMEM with the same virus stabilizer contained in the vaccine (vegetable-derived carbohydrates and amino acids). The placebo was identical in appearance to the dengue vaccine.	

DENV = dengue virus; FFU = focus forming unit.

All vaccine lots and placebo were produced at the U.S. Army WRAIR Pilot Bioproduction Facility in Silver Spring, MD.

Immunofocus assay testing was performed 22 months post-manufacture and prior to the first vaccination date.

**Table 2:** Number and percentage of subjects with solicited AEs reported during the 21-day (days 0–20) postvaccination periods based on priming status (TVC)

		Any solicited AE		Any solicited general AE		Any solicited injection site AE	
	Group (*N*)	%	95% CI	%	95% CI	%	95% CI
All subjects	F17 (207)	72.0	(65.3–78.0)	66.2	(59.3–72.6)	25.1	(19.4–31.6)
	F19 (211)	69.7	(63.0–75.8)	64.9	(58.1–71.4)	28.4	(22.5–35.0)
	Placebo (210*)	65.7	(58.9–72.1)	61.0	(54.0–67.6)	21.9	(16.5–28.1)
Unprimed	F17 (104)	75.0	(65.6–83.0)	70.2	(60.4–78.8)	26.0	(17.9–35.5)
	F19 (102)	73.5	(63.9–81.8)	69.6	(59.7–78.3)	28.4	(19.9–38.2)
	Placebo (90)	61.1	(50.3–71.2)	56.7	(45.8–67.1)	18.9	(11.4–28.5)
Primed	F17 (103)	68.9	(59.1–77.7)	62.1	(52.0–71.5)	24.3	(16.4–33.7)
	F19 (109)	66.1	(56.4–74.9)	60.6	(50.7–69.8)	28.4	(20.2–37.9)
	Placebo (118)	69.5	(60.3–77.6)	64.4	(55.1–73.0)	24.6	(17.1–33.4)

AE = adverse event; 95% CI = exact 95% confidence interval (lower and upper limits); *N* = number of subjects with at least one dose injected and accompanied by a completed diary card; % = percentage of subjects presenting at least one type of symptoms; TVC = total vaccinated cohort.

* Unknown priming status for two subjects in the placebo group.

**Table 3:** Number and percentage of subjects with solicited injection site reactions reported during the 21-day (days 0–20) postvaccination periods (TVC)

Injection site reaction		F17 *N* = 207		F19 *N* = 211		Placebo *N* = 210	
	Type	*n*	(95% CI)	*n*	(95% CI)	*n*	(95% CI)
Pain	All	44	21.3 (15.9, 27.5)	53	25.1 (19.4, 31.5)	41	19.5 (14.4, 25.5)
	Grade 3	1	0.5 (0.0, 2.7)	0	0.0 (0.0, 1.7)	0	0.0 (0.0, 1.7)
Redness	All	23	11.1 (7.2, 16.2)	20	9.5 (5.9, 14.3)	16	7.6 (4.4, 12.1)
	Grade 3	5	2.4 (0.8, 5.5)	2	0.9 (0.1, 3.4)	4	1.9 (0.5, 4.8)
Swelling	All	16	7.7 (4.5, 12.2)	13	6.2 (3.3, 10.3)	9	4.3 (2.0, 8.0)
	Grade 3	3	1.4 (0.3, 4.2)	0	0.0 (0.0, 1.7)	1	0.5 (0.0, 2.6)

All = any intensity of injection site reaction; 95% CI = exact 95% confidence interval (lower and upper limit); Grade 3 pain = pain that prevented normal, everyday activities; Grade 3 redness/swelling = diameter of redness/swelling > 20 mm; *N* = number of subjects with at least one dose injected and accompanied by a completed diary card; *n*/% = number/percentage of subjects reporting the injection site reaction; TVC = total vaccinated cohort.

**Table 4:** Number and percentage of subjects with solicited general AEs reported during the 21-day (days 0–20) postvaccination periods (TVC)

		F17 *N* = 207		F19 *N* = 211		Placebo *N* = 210	
AE	Type	*n*	(95% CI)	*n*	(95% CI)	*n*	(95% CI)
Abdominal pain	All	27	13.0 (8.8, 18.4)	35	16.6 (11.8, 22.3)	24	11.4 (7.5, 16.5)
	Grade 3	0	0.0 (0.0, 1.8)	1	0.5 (0.0, 2.6)	1	0.5 (0.0, 2.6)
Fatigue	All	24	11.6 (7.6, 16.8)	32	15.2 (10.6, 20.7)	27	12.9 (8.6, 18.2)
	Grade 3	0	0.0 (0.0, 1.8)	1	0.5 (0.0, 2.6)	0	0.0 (0.0, 1.7)
Fever	All	81	39.1 (32.4, 46.1)	81	38.4 (31.8, 45.3)	70	33.3 (27.0, 40.1)
	Grade 3	10	4.8 (2.3, 8.7)	13	6.2 (3.3, 10.3)	8	3.8 (1.7, 7.4)
Headache	All	62	30.0 (23.8, 36.7)	60	28.4 (22.5, 35.0)	58	27.6 (21.7, 34.2)
	Grade 3	2	1.0 (0.1, 3.4)	1	0.5 (0.0, 2.6)	6	2.9 (1.1, 6.1)
Pruritus	All	25	12.1 (8.0, 17.3)	25	11.8 (7.8, 17.0)	18	8.6 (5.2, 13.2)
	Grade 3	0	0.0 (0.0, 1.8)	0	0.0 (0.0, 1.7)	1	0.5 (0.0, 2.6)
Rash	All	17	8.2 (4.9, 12.8)	19	9.0 (5.5, 13.7)	10	4.8 (2.3, 8.6)
	Grade 3	0	0.0 (0.0, 1.8)	0	0.0 (0.0, 1.7)	1	0.5 (0.0, 2.6)
Vomiting	All	22	10.6 (6.8, 15.6)	23	10.9 (7.0, 15.9)	20	9.5 (5.9, 14.3)
	Grade 3	0	0.0 (0.0, 1.8)	1	0.5 (0.0, 2.6)	1	0.5 (0.0, 2.6)

AE = adverse event; All = any intensity of AE; 95% CI = exact 95% confidence interval (lower and upper limits); Fever = oral body temperature ≥ 37.5°C, Fever Grade 3 > 39.0°C; Grade 3 = an AE that prevented normal, everyday activities; *N* = number of subjects with at least one dose injected and accompanied by a completed diary card; *n*/% = number/ percentage of subjects reporting the AE at least once; TVC = total vaccinated cohort.

**Table 5:** Seropositivity rates for neutralizing antibody to each DENV type based on priming status (ATP cohort for immunogenicity)

ATP cohort, primed, and unprimed subjects: *N* = 507						
			Unprimed subjects		Primed Subjects	
Antibody	Group	Timing	*N*	% ≥ 1:10 (95% CI)	*N*	% ≥ 1:10 (95% CI)
DENV-1	F17	PRE	86	0.0 (0.0–4.2)	91	97.8 (92.3–99.7)
		PI(M3)	83	20.5 (12.4–30.8)	88	97.7 (92.0–99.7)
		PII(M7)	82	95.1 (88.0–98.7)	87	100 (95.8–100)
	F19	PRE	61	0.0 (0.0–5.9)	94	96.8 (91.0–99.3)
		PI(M3)	60	26.7 (16.1–39.7)	93	98.9 (94.2–100)
		PII(M7)	59	83.1 (71.0–91.6)	90	100 (96.0–100)
DENV-2	F17	PRE	86	0.0 (0.0–4.2)	91	97.8 (92.3–99.7)
		PI(M3)	83	26.5 (17.4–37.3)	88	96.6 (90.4–99.3)
		PII(M7)	82	89.0 (80.2–94.9)	87	100 (95.8–100)
	F19	PRE	61	0 (0.0–5.9)	94	96.8 (91.0–99.3)
		PI(M3)	60	43.3 (30.6–56.8)	93	98.9 (94.2–100)
		PII(M7)	59	93.2 (83.5–98.1)	90	98.9 (94.0–100)
DENV-3	F17	PRE	86	0.0 (0.0–4.2)	91	96.7 (90.7–99.3)
		PI(M3)	83	14.5 (7.7–23.9)	88	96.6 (90.4–99.3)
		PII(M7)	82	92.7 (84.8–97.3)	87	100 (95.8–100)
	F19	PRE	61	0.0 (0.0–5.9)	94	92.6 (85.3–97.0)
		PI(M3)	60	21.7 (12.1–34.2)	93	98.9 (94.2–100)
		PII(M7)	59	78.0 (65.3–87.7)	90	98.9 (94.0–100)
DENV-4	F17	PRE	86	0.0 (0.0–4.2)	91	96.7 (90.7–99.3)
		PI(M3)	83	37.3 (27.0–48.7)	88	98.9 (93.8–100)
		PII(M7)	82	97.6 (91.5–99.7)	87	100 (95.8–100)
	F19	PRE	61	0.0 (0.0–5.9)	94	98.9 (94.2–100)
		PI(M3)	60	26.7 (16.1–39.7)	93	98.9 (94.2–100)
		PII(M7)	59	86.4 (75.0–94.0)	90	98.9 (94.0–100)

ATP = according to protocol; 95% CI = exact 95% confidence interval (lower and upper limits); DENV = dengue virus; *N* = number of subjects with available results; *n*/% = number/percentage of subjects with titer > = 1:10; PI(M3) = post-dose 1, Month 3 visit; PII(M7) = post-dose 2, Month 7 visit; PRE = pre-vaccination.

**Table 6:** GMTs for neutralizing antibody to each DENV type in dengue unprimed subjects (ATP cohort for immunogenicity)

ATP cohort, unprimed subjects: *N* = 221				
Antibody	Group	Timing	*N*	GMT (95% CIs)
DENV-1	F17	PRE	86	5.0 (5.0, 5.0)
		PI(M3)	83	7.9 (6.2, 10.0)
		PII(M7)	82	130.9 (92.8, 184.7)
	F19	PRE	61	5.0 (5.0,5.0)
		PI(M3)	60	9.2 (6.7, 12.6)
		PII(M7)	59	84.4 (50.5, 141.0)
DENV-2	F17	PRE	86	5.0 (5.0, 5.0)
		PI(M3)	83	12.1 (8.3, 17.7)
		PII(M7)	82	218.6 (141.7, 337.2)
	F19	PRE	61	5.0 (5.0, 5.0)
		PI(M3)	60	26.3 (14.6, 47.2)
		PII(M7)	59	215.2 (133.8, 346.0)
DENV-3	F17	PRE	86	5.0 (5.0, 5.0)
		PI(M3)	83	7.2 (5.7, 9.1)
		PII(M7)	82	124.5 (85.5, 181.2)
	F19	PRE	61	5.0 (5.0, 5.0)
		PI(M3)	60	7.6 (5.9, 9.7)
		PII(M7)	59	60.3 (37.7, 96.3)
DENV-4	F17	PRE	86	5.0 (5.0, 5.0)
		PI(M3)	83	17.6 (11.2, 27.5)
		PII(M7)	82	377.7 (273.3, 521.8)
	F19	PRE	61	5.0 (5.0, 5.0)
		PI(M3)	60	11.0 (7.2, 16.6)
		PII(M7)	59	105.9 (64.4, 174.2)

ATP = according to protocol; 95% CI = 95% confidence intervals; DENV = dengue virus; GMT = geometric mean antibody titer calculated on all subjects; *N* = number of subjects with available results; PI(M3) = post-dose 1 Month 3 visit; PII(M7) = post-dose 2 Month 7 visit; PRE = pre-vaccination.

**Table 7:** GMTs for neutralizing antibody to each DENV type in primed subjects (ATP cohort for immunogenicity)

ATP cohort, primed subjects: *N* = 286				
Antibody	Group	Timing	*N*	GMT (95% CIs)
DENV-1	F17	PRE	91	564.3 (409.4, 777.8)
		PI(M3)	88	1161.6 (896.4, 1505.1)
		PII(M7)	87	1412.8 (1191.5, 1675.3)
	F19	PRE	94	448.5 (311.7, 645.4)
		PI(M3)	93	1283.2 (1023.6, 1608.5)
		PII(M7)	90	1324.1 (1104.1, 1587.9)
DENV-2	F17	PRE	91	529.1 (374.2, 748.2)
		PI(M3)	88	1290.3 (991.5, 1679.2)
		PII(M7)	87	1514.4 (1322.2, 1734.6)
	F19	PRE	94	448.2 (320.1, 627.4)
		PI(M3)	93	1590.1 (1327.8, 1904.2)
		PII(M7)	90	1338.5 (1105.6, 1620.6)
DENV-3	F17	PRE	91	499.1 (351.3, 709.0)
		PI(M3)	88	1080.7 (802.2, 1456.1)
		PII(M7)	87	1130.0 (920.0, 1388.0)
	F19	PRE	94	323.5 (217.3, 481.6)
		PI(M3)	93	1167.7 (921.7, 1479.4)
		PII(M7)	90	901.4 (707.6, 1148.3)
DENV-4	F17	PRE	91	452.2 (320.7, 637.7)
		PI(M3)	88	1041.7 (800.5, 1355.7)
		PII(M7)	87	1228.4 (1014.1, 1488.0)
	F19	PRE	94	349.2 (245.1, 497.5)
		PI(M3)	93	1147.5 (908.5, 1449.3)
		PII(M7)	90	1020.8 (809.5, 1287.4)

ATP = according to protocol; 95% CI = 95% confidence intervals; DENV = dengue virus; GMT = geometric mean antibody titer calculated on all subjects; *N* = number of subjects with available results; PI(M3) = post-dose 1 Month 3 time point; PII(M7) = post-dose 2 Month 7 time point; PRE = pre-vaccination.
