# Supplementary material for: A Phase II, Randomized, Safety and Immunogenicity Trial of a Re-Derived, Live-Attenuated Dengue Virus Vaccine in Healthy Children and Adults Living in Puerto Rico
Source: Am J Trop Med Hyg. 2015 Sep 2;93(3):441–53. doi: 10.4269/ajtmh.14-0625 (PMC4559678; doi:10.4269/ajtmh.14-0625)
Supplement: Supplementary file 1 [file SD1.pdf]

Unprimed subjects

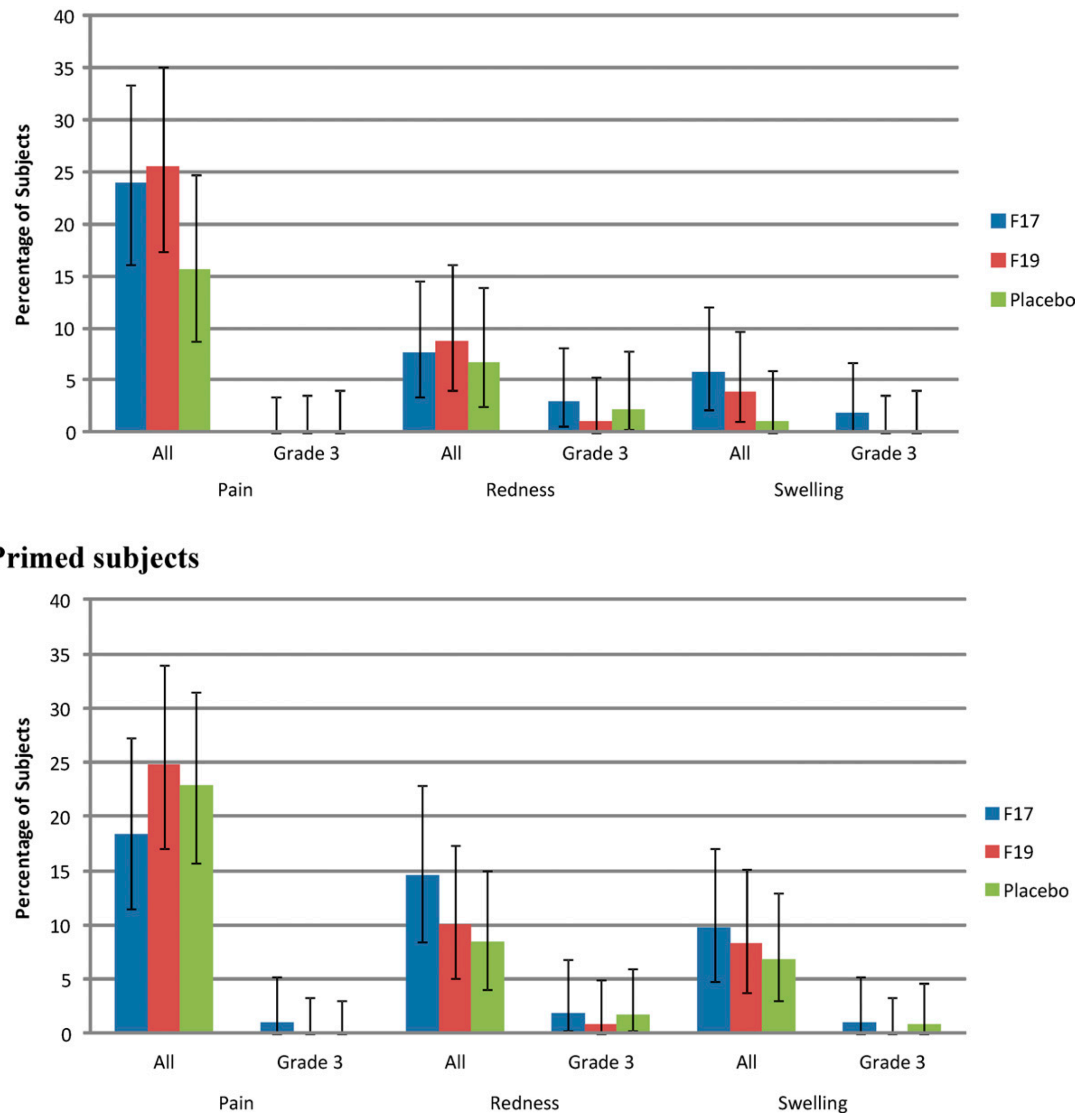

Supplemental Figure 1. Percentage of subjects with solicited injection site reactions reported during the 21-day (days 0–20) post-vaccination periods based on priming status (TVC).

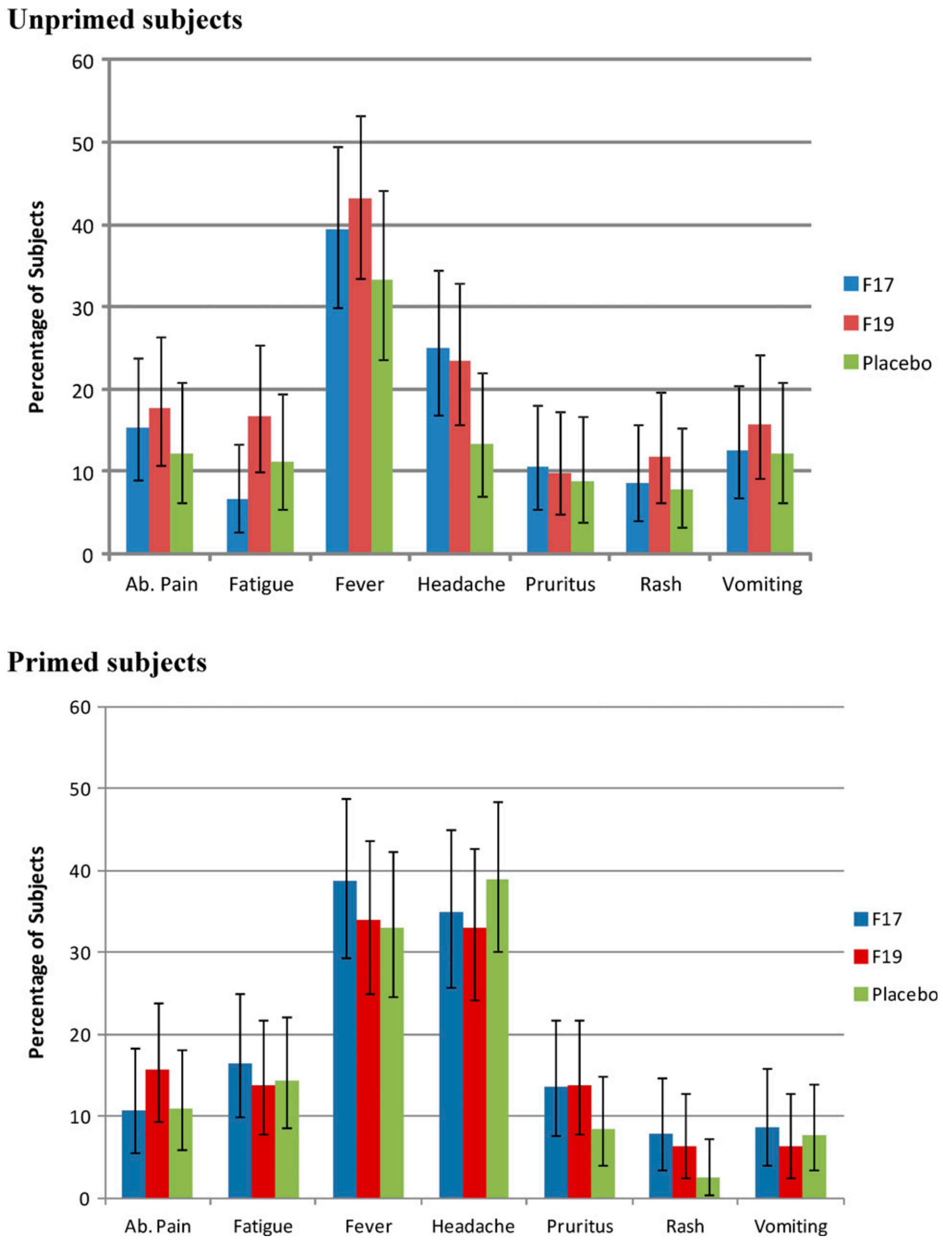

SUPPLEMENTAL FIGURE 2. Percentage of subjects with solicited general AEs reported during the 21-day (days 0–20) post-vaccination periods based on priming status (TVC).

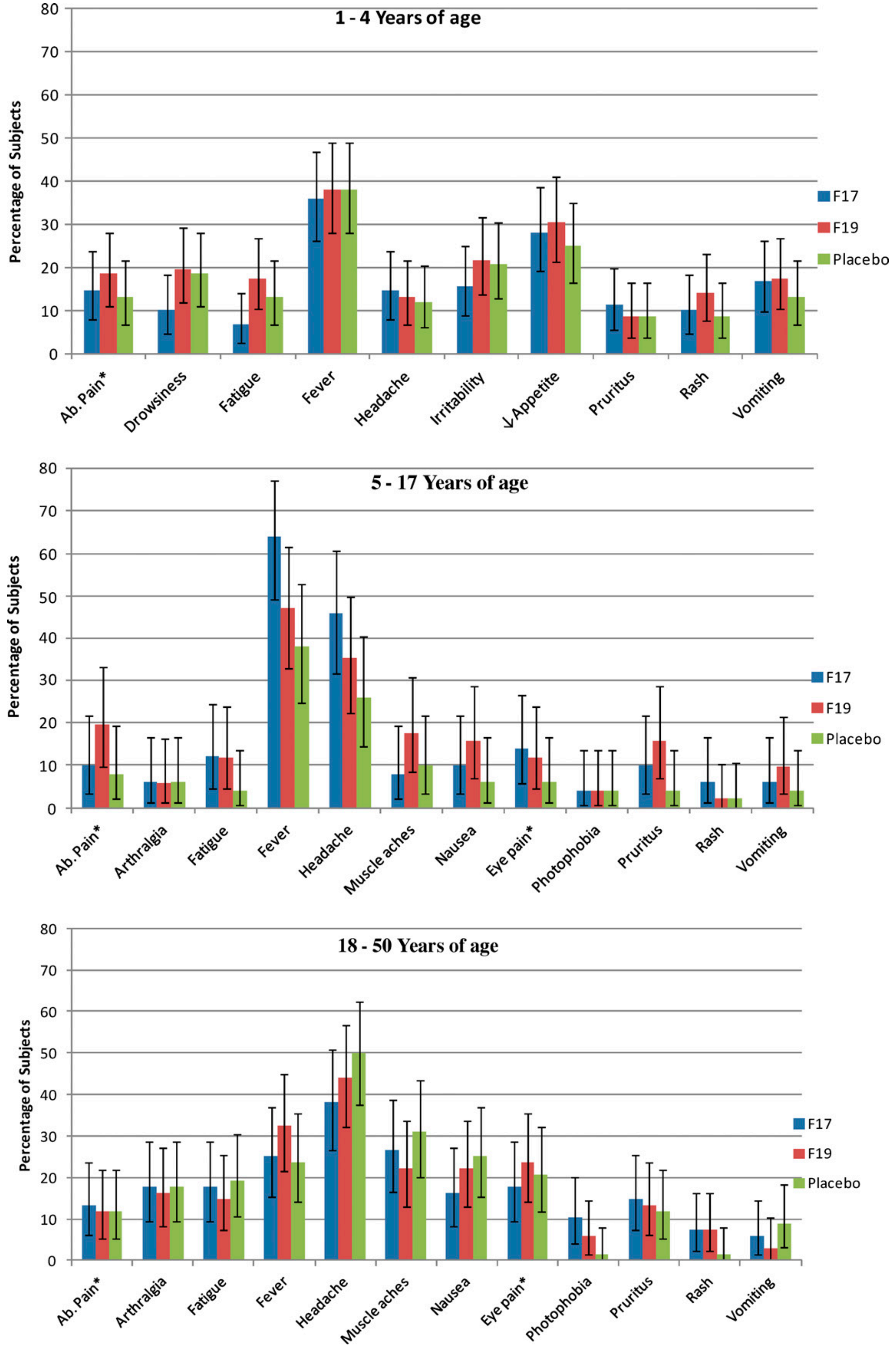

Supplemental Figure 3. Solicited general AEs (overall per subject) reported during the 21-day post-vaccination based on age (TVC). * Ab. Pain = abdominal pain; eye pain = pain behind the eyes. ↓ Appetite = loss of appetite.
